# Simultaneous analysis of urinary total 4-(methylnitrosamino)-1-(3-pyridyl)-1-butanol, *N*′-nitrosonornicotine, and cotinine by liquid chromatography-tandem mass-spectrometry

**DOI:** 10.1038/s41598-021-99259-z

**Published:** 2021-10-08

**Authors:** Sampada S. Nikam, Murari Gurjar, Hitesh Singhavi, Anand Patil, Arjun Singh, Peter Villalta, Pankaj Chaturvedi, Samir S. Khariwala, Vikram Gota, Irina Stepanov

**Affiliations:** 1 Department of Clinical Pharmacology, Advanced Centre for Training, Research and Education in Cancer (ACTREC), Mumbai, India; 2 Tata Memorial Centre, Mumbai, India; 3 Masonic Cancer Center, University of Minnesota, 2231 6th Street SE, Minneapolis, MN 55455 USA; 4 Department of Otolaryngology, Head and Neck Surgery, Medical School, University of Minnesota, Minneapolis, USA; 5 Division of Environmental Health Sciences, School of Public Health, University of Minnesota, Minneapolis, USA; 6 Homi Bhabha National Institute, Mumbai, India

**Keywords:** Mass spectrometry, Biochemistry, Biomarkers

## Abstract

Biomarkers of exposure to harmful tobacco constituents are key tools for identifying individuals at risk and developing interventions and tobacco control measures. However, tobacco biomarker studies are scarce in many parts of the world with high prevalence of tobacco use. Our goal was to establish a robust method for simultaneous analysis of urinary total 4-(methylnitrosamino)-1-(3-pyridyl)-1-butanol (NNAL), *N*′-nitrosonornicotine (NNN), and cotinine at the Advanced Centre for Treatment, Research and Education in Cancer (ACTREC) in Mumbai, India. These biomarkers are validated measures of exposure to the carcinogenic tobacco nitrosamines 4-(methylnitrosamino)-1-(3-pyridyl)-1-butanone (NNK) and NNN and the addictive alkaloid nicotine, respectively. The established method is characterized by excellent accuracy, linearity, and precision, and was successfully applied to the analysis of 15 smokeless tobacco (SLT) users and 15 non-users of tobacco recruited in Mumbai. This is the first report of establishment of such procedure in a laboratory in India, which offers the first in-country capacity for research on tobacco carcinogenesis in Indian SLT users.

## Introduction

Tobacco use remains the leading preventable cause of morbidity and mortality globally, accounting for 8 million annual deaths worldwide^1^. While steady declines in smoking prevalence have been registered over the last decades in some high-income countries, the prevalence of tobacco use in many parts of the world remains unchanged or even increasing. For example, use of smokeless tobacco (SLT) products remains high in India and other Southeast Asian countries^2–4^, and smoking is on the rise in several countries with low-income economies that became targets for cigarette advertising by transnational tobacco companies^5^.

Research employing biomarkers of toxic and carcinogenic tobacco constituents can provide important insights for identifying individuals at risk and developing interventions and tobacco control measures. Biomarkers of nicotine, 4-(methylnitrosamino)-1-(3-pyridyl)-1-butanone (NNK), and *N*′-nitrosonornicotine (NNN) are of particular importance because these constituents are specific to tobacco and play important roles in tobacco-associated health outcomes. Nicotine is the major known addictive constituent in tobacco, and its biomarker cotinine has been historically used as a measure of tobacco product consumption^6,7^. NNK and NNN are potent organ-specific carcinogens in laboratory animals: NNK causes lung cancer independent of the route of administration, and NNN causes cancers of oral cavity and esophagus^2,8,9^. In tobacco users, exposure to NNK can be measured via its biomarker urinary total 4-(methylnitrosamino)-1-(3-pyridyl)-1-butanol (NNAL) and exposure to NNN is measured via urinary total NNN^10–12^. Epidemiological studies in smokers showed that levels of urinary total NNAL and NNN are prospectively associated with the risk for developing lung cancer^13,14^ and esophageal cancer^15,16^, respectively. In the United States, studies assessing cotinine, total NNAL, and total NNN in tobacco users generated important evidence to support tobacco control measures such as clean air laws and the proposal by the Food and Drug Administration to regulate levels of NNN in SLT products to protect public health^17^. However, because of the lack of expertise and resources, tobacco biomarker studies are scarce in parts of the world that could benefit the most from such research.

The overarching goal of our research efforts is to develop capacity for tobacco biomarker research in India. Here we report a method for simultaneous analysis of cotinine, NNAL and NNN in human urine, which was optimized at the Advanced Centre for Treatment, Research and Education in Cancer (ACTREC) in Mumbai, India. Previously reported methods measured either a single biomarker or a combination of two biomarkers^18–22^. In our ongoing and future research efforts in India, we aim to analyze all three biomarkers in large numbers of urine samples in order to characterize the links between the chemical composition of SLT and other tobacco products and the exposures and health outcomes in users. The ability to analyze all three biomarkers in one assay is essential for accelerating such research. Furthermore, simultaneous analysis of these three important tobacco biomarkers offers a cost-effective solution for conducting tobacco control research in low-resource settings.

## Results

### Development and optimization of the assay

**Figure 1:**
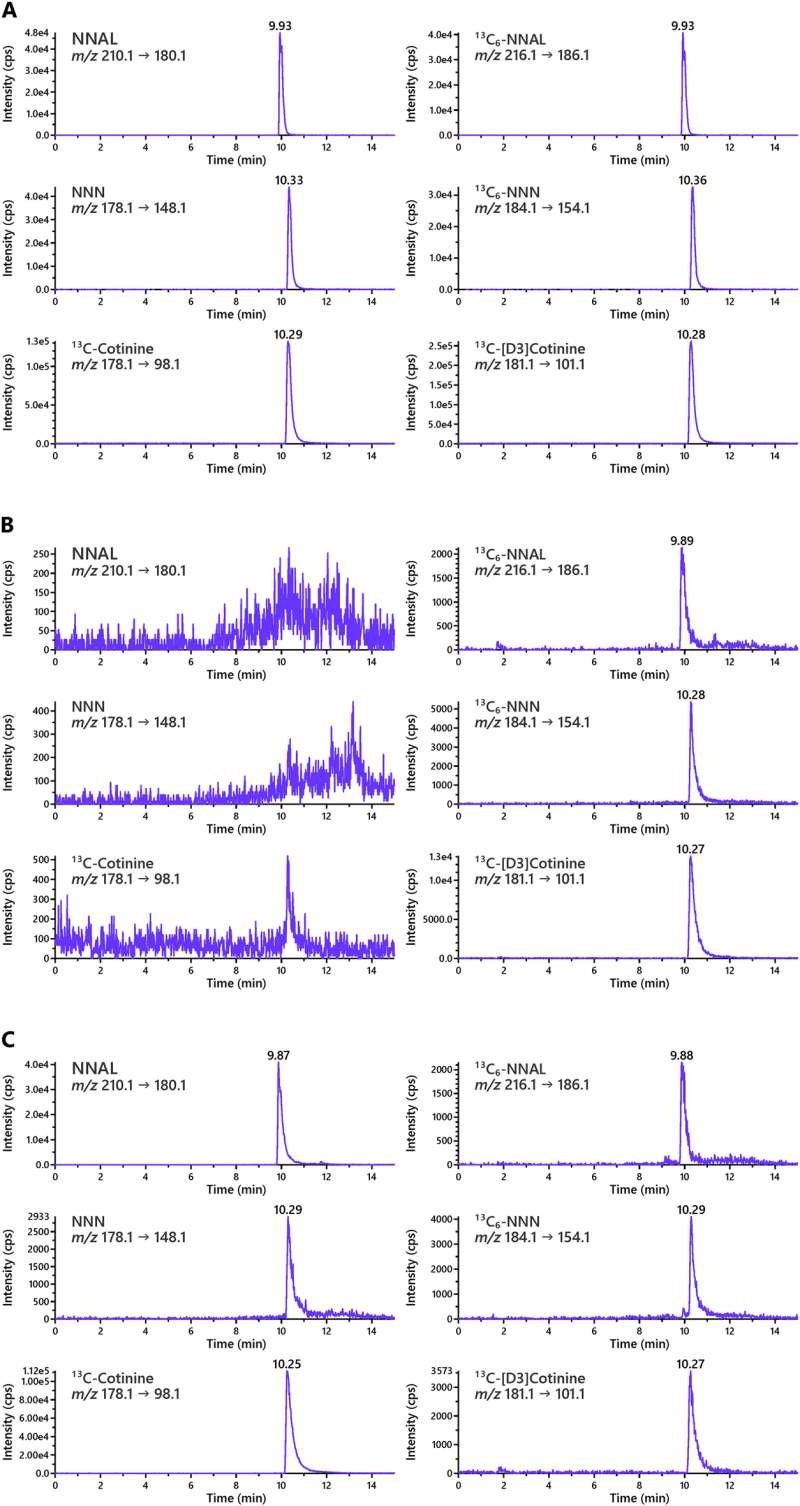
Representative LC–MS/MS chromatograms of NNAL, NNN and cotinine (left panel) and their respective internal standards (right panel) in (**A**) standard mix; (**B**) urine sample from a non-user of tobacco; and (**C**) urine sample from an SLT user.

The liquid chromatography-tandem mass spectrometry (LC–MS/MS) method for the analysis of NNAL, NNN, and cotinine in the purified urine samples was essentially based on the previously reported methods^21–25^, except that a non-capillary system and a QTRAP instrument were used in this study. Given the higher flow rate used in this system, we optimized the HPLC mobile phase gradient to allow for chromatographic resolution of analytes from the potential peaks of residual interfering metabolites that may be present in the complex urine matrix. Representative chromatograms obtained upon LC–MS/MS analysis of a standard mix containing NNAL, NNN, and cotinine and corresponding labeled internal standards [^13^C_6_]NNAL, ^13^C_6_NNN, and [D_3_]cotinine are shown in Fig. 1A.

**Figure 2:**
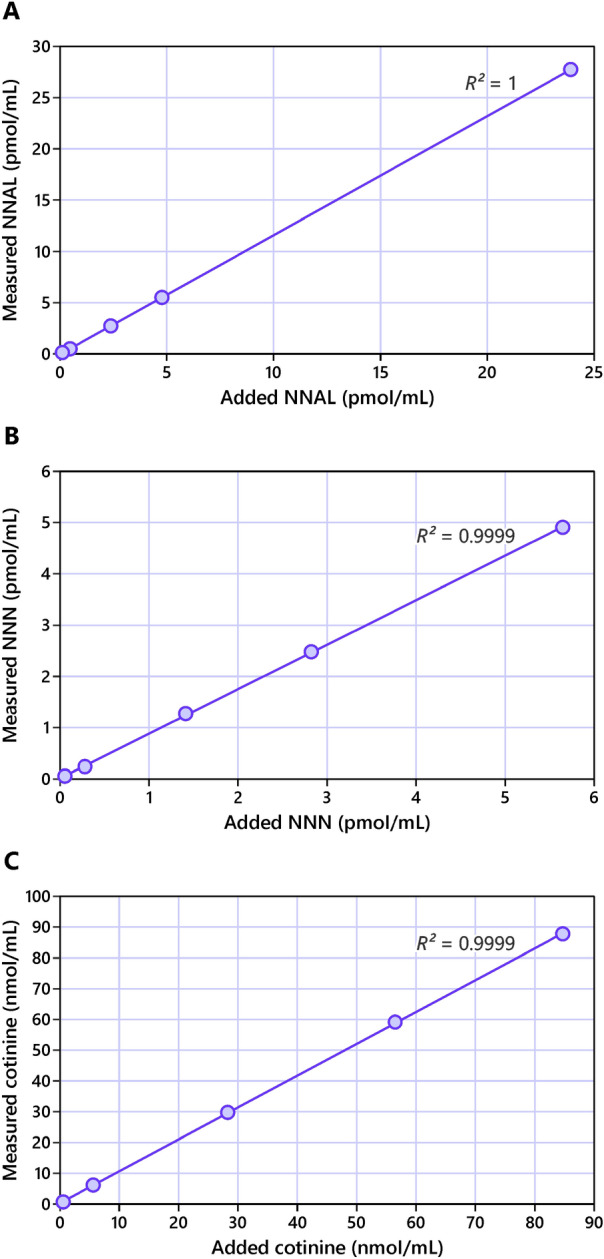
Relationship between added and measured concentrations of (**A**) NNAL, (**B**) NNN, and (**C**) cotinine. The biomarkers were added to a pooled urine from non-users of tobacco. For more detail on method characterization see “Experimental procedures” section.

The method reported here also includes a newly developed sample purification procedure to allow for simultaneous extraction and enrichment of all three biomarkers, which distinguishes it from the previously published assays for urinary total NNAL, NNN, and cotinine^19–22^. The characteristics of the method are summarized in Table 1. Accuracy of the assay (added vs. measured) in pooled non-tobacco user urine averaged 110.9% for total NNAL, 89.2% for total NNN, and 107.2% for cotinine. Precision (coefficient of variation, CV) of measurements at the lowest levels of addition were 4.2% for NNAL, 13.8% for NNN, and 2.6% for cotinine (Supplemental Table S1). Linearity of the method in the assessed range of concentrations is illustrated in Fig. 2.

**Table 1:** Method characteristics for simultaneous analysis of urinary total NNAL, NNN and cotinine.

Characteristic	Biomarker^a^		
	NNAL	NNN	Cotinine
Accuracy^b^, %	110.9 (102.5–115.3)	89.2 (85.1–96.4)	107.2 (103.5–113.3)
Precision^b^, %	2.9 (0.5–7.2)	4.9 (1.5–13.8)	1.9 (0.8–2.6)
Intra-day variation, % (N = 6)	10.3	7.0	8.6
Inter-day variation, % (N = 12)	10.2	11.2	9.5

^a^Biomarker abbreviations: NNAL, 4-(methylnitrosamino)-1-(3-pyridyl)-1-butanol; NNN, *N*′-nitrosonornicotine.

^b^Accuracy and precision were determined in pooled urine from non-users of tobacco to which biomarkers were added in the ranges from 0.1 to 23.9 pmol/mL for NNAL, 0.1 to 5.6 pmol/mL for NNN, and 0.6 to 84.7 nmol/mL for cotinine. The numbers shown are accuracy and precision averages for all levels of addition and minimum–maximum ranges (in parentheses). For more detail on method characterization see “Methods” section.

The performance of the method was assessed by using the positive control urine data (pooled smokers’ urine) that is being routinely used as a quality control for tobacco biomarker analyses in Dr. Stepanov’s laboratory at University of Minnesota. The levels of NNAL, NNN, and cotinine measured in the positive control urine by the developed method were 1.3 (± 0.01) pmol/mL, 0.055 (± 0.1) pmol/mL, and 20 (± 1.8) nmol/mL, respectively. These levels were comparable with the values measured in the same positive control stock in Dr. Stepanov’s laboratory: 1.4 (± 0.20) pmol/mL, 0.052 (± 0.005) pmol/mL, and 22.5 (± 2.1) nmol/mL, respectively. Intra- and inter-day variations for biomarker measurement in these samples, as analyzed by the developed method, are shown in Table 1.

### Analysis of urine samples from SLT users and non-users of tobacco products

The method was applied to the analysis of 30 urine samples: 15 SLT users and 15 non-users of tobacco. Water blanks, which were used as negative controls, did not contain detectable levels of any of the biomarkers. Typical chromatograms obtained upon analysis of urine samples are illustrated in Fig. 1B,C. Levels of urinary total NNAL, NNN and cotinine in all subjects are summarized in Table 2. The limit of quantitation (LOQ, determined at signal-to noise ratio of 5) for total NNAL was 0.05 pmol/mL, for total NNN 0.01 pmol/mL, and for cotinine 0.05 nmol/mL. All SLT users had quantifiable total NNAL, NNN and cotinine in their urine. Levels of urinary total NNAL, NNN, and cotinine in these subjects averaged 3.67 (± 5.8) pmol/mL, 0.18 (± 0.3) pmol/mL, and 14.33 (± 13.9) nmol/mL, respectively. In non-users of tobacco, total cotinine was present in urine of 12 out of 15 subjects, ranging from LOQ to 0.07 nmol/mL, while total NNAL and total NNN were non-detectable.

**Table 2:** Total NNAL, NNN, and cotinine in urine samples from smokeless tobacco users and non-users of tobacco recruited in Mumbai, India.

Individual subjects	Biomarker levels		
	Total NNAL (pmol/mL)	Total NNN (pmol/mL)	Total cotinine (nmol/mL)
**Smokeless tobacco users**			
1	0.75	0.02	13.3
2	0.71	0.05	5.5
3	2.22	0.14	11.1
4	1.41	0.04	12.2
5	0.96	0.03	8.5
6	1.56	0.05	4.3
7	0.60	0.02	3.6
8	22.6	1.35	44.2
9	0.76	0.02	2.9
10	8.27	0.34	48.3
11	1.17	0.02	5.1
12	0.32	0.04	7.2
13	5.39	0.27	17.9
14	6.30	0.16	19.3
15	2.05	0.17	11.5
Mean ± SD	3.67 ± 5.8	0.18 ± 0.3	14.33 ± 13.9
**Non-users of tobacco**			
1	ND	ND	LOQ
2	ND	ND	0.06
3	ND	ND	0.02
4	ND	ND	0.07
5	ND	ND	0.02
6	ND	ND	0.02
7	ND	ND	ND
8	ND	ND	ND
9	ND	ND	0.02
10	ND	ND	0.01
11	ND	ND	0.01
12	ND	ND	0.01
13	ND	ND	0.01
14	ND	ND	0.01
15	ND	ND	ND
Mean ± SD	N/A	N/A	0.03 ± 0.02

Abbreviations: NNAL, 4-(methylnitrosamino)-1-(3-pyridyl)-1-butanol; NNN, N′-nitrosonornicotine, ND, not detected; LOQ, limit of quantitation; N/A, not applicable.

## Discussion

Analysis of tobacco biomarkers is an important tool in studies of risks associated with tobacco use. We present here the development of the procedure for the analysis of urinary total NNAL, NNN, and cotinine, to achieve simultaneous measurement of these important biomarkers in a resource-efficient way. This is the first report of establishment of such procedure in a laboratory in Mumbai, India, which offers the first in-country capacity for research on tobacco carcinogenesis in Indian SLT users. While the majority of published studies on the analysis of urinary total NNAL and NNN used low-flow capillary HPLC coupled with triple-quadrupole mass-spectrometry detectors^21–25^, the assay reported here utilizes a non-capillary, low-flow HPLC and AB Sciex QTRAP system. Such analytical equipment is widely used for robust analyses of drug metabolites in pharmacokinetics studies worldwide^26,27^ and therefore is more likely to be available in countries where the procurement of dedicated mass-spectrometry equipment for tobacco biomarker research may not be feasible due to limited resources.

Our reported method leverages the previously developed approach that takes advantage of the presence of ^13^C-cotinine in urine and monitors the mass-spectrometry signal for its [M + H]^+^ ion with *m/z* 178.1, which accounts for only 11% of the ^12^C-cotinine [M + H]^+^ signal^21^. Because levels of cotinine in urine of tobacco users are several thousand-fold higher than those of total NNAL and total NNN, such reduction in the cotinine signal is key to avoiding the ion source or detector saturation and the resulting challenges in simultaneous accurate quantitation of all three biomarkers^28^. In addition, to achieve simultaneous purification of NNAL and cotinine with NNN, it was necessary to increase the strength of the elution solvent during the normal-phase extraction, which is an important step in sample purification for urinary total NNN analysis^20,23,25^. Addition of 10% MeOH allowed for removal of more polar NNAL and cotinine from the Silica Bond Elut cartridges together with NNN, which normally can be removed with 100% EtOAc. We note that a combined assay of urinary NNAL and NNN with the use of normal-phase solid phase extraction has been previously proposed^22^. However, this is the first report of the method for simultaneous analysis of all three biomarkers.

We used ascorbic acid to prevent artefactual formation of NNN via nitrosation of nornicotine which is present in urine of tobacco users^23,29,30^. Urine also contains nitrates and nitrites, and sample processing exposes samples to nitrogen oxides in the air, all of which can produce nitrosating species capable of reacting with nornicotine to form NNN^30,31^. Ascorbic acid acts as a scavenger of such nitrosating species, and we previously used its addition to prevent nornicotine nitrosation and artefactual NNN formation during sample processing^23,32,33^. Similar approach with ammonium sulfamate as the inhibitor of nitrosation was also used in the published method for combined NNAL and NNN measurement^22^. Our method is different in that we add ascorbic acid prior to purification of urine samples by MCX, instead of after such purification. This assures that NNN formation does not take place when sample is acidified prior to loading on MCX cartidges^31^, and preserves the purity of samples after the desalting that occurs during MCX purification. We previously employed the addition of [pyridine-D_4_]nornicotine to biological samples and subsequent monitoring of [pyridine-D_4_]NNN to demonstrate that ascorbic acid, as used here, prevents artefactual NNN formation^23^.

The optimized method is characterized by excellent accuracy, linearity, and precision (Table 1, Fig. 2). We note that method accuracy for NNN was somewhat lower than for other biomarkers (Fig. 1C). This is potentially due to lower levels of this biomarker compared to other two, and higher background noise resulting from interfering polar compounds present in the residual sample matrix. The original method for separate analysis of total NNN does not add 10% MeOH to the elution solvent at the last step of normal-phase extraction, which facilitates the removal of interfering polar metabolites, reduces the background noise, and improves the accuracy of NNN assay. However, the accuracy for NNN in the combined procedure reported here is still in the acceptable range for analytical assays (80–120% per U.S. FDA guidelines for bioanalytical method validation).

We applied the developed method to the analysis of a subset of urine samples that are being collected as part of our on-going research on tobacco carcinogenesis in Mumbai (Table 2). In the urine of SLT users, levels of NNAL and NNN were comparable to, and cotinine levels were lower than, those reported for smokers and SLT users in the US^20,21,24,34^. It is important to note that 10 out of 15 SLT users reported using *gutkha* and *betel quid*—SLT products in India that contain only small amounts of tobacco mixed with other ingredients^35,36^. Many other popular SLT products in India contain extremely high levels of NNN, NNK, and nicotine^37,38^, and exposures in users of such products are expected to be significantly higher than in smokers. Efforts are underway to analyze urine samples collected from users of various SLT products. In non-users of tobacco, only cotinine was present at trace levels, while total NNAL and total NNN were below LOD (Table 2). Exposures to secondhand cigarette smoke or to environmental residues of smokeless tobacco could be responsible for the presence of cotinine in the urine of non-users of tobacco products. The trace levels of cotinine in some non-users are likely due to exposures to secondhand cigarette smoke (SHS) or to environmental residues of SLT. It is important to note that SHS exposure is also a source of exposure to NNK and NNN; however, levels of these constituents in tobacco products are much lower than those of nicotine. Indeed, previous studies of SHS exposure in adults report a range of 0.33 × 10^3^–1.04 × 10^3^ for the ratio of urinary total NNAL to cotinine, with some individuals having no detectable NNAL even if cotinine is present^39^. Lastly, due to low prevalence of smoking, the frequency of exposure to SHS is expected to be low in India. Future studies aimed at characterizing SHS or other environmental exposures to NNK and NNN in non-tobacco users in India should use larger starting volumes of urine to assure accurate quantitation of respective urinary biomarkers.

In summary, we report here a robust and resource-effective method for simultaneous analysis of three important tobacco-specific biomarkers in human urine. The method has been established in the analytical laboratory at ACTREC in Mumbai, India, creating a vital resource for conducting tobacco carcinogenesis research in this country. Future efforts are warranted to adapt this method to 96-well format to facilitate tobacco biomarker measurements in large epidemiological studies. Such research will serve to support tobacco control efforts in India and potentially in other countries in Southeast Asia.

## Methods

### Caution

NNAL and NNN are carcinogenic and mutagenic and should be handled with extreme care, using appropriate protective clothing and ventilation at all times.

### Materials and reagents

Analytical standards for NNAL, NNN, cotinine and their respective isotope-labeled analogues [^13^C_6_]NNAL, ^13^C_6_NNN, and [D_3_]cotinine were obtained from Toronto Research Chemicals, Inc. (Canada). *β*-Glucuronidase (Type IX from *E. coli*) was purchased from Sigma Aldrich Chemicals (Bangalore, India). Diatomaceous Earth Chem Elut (5 mL unbuffered) cartridges and Bond Elut 1 cc (100 mg, LRC-SI) cartridges were purchased from Agilent Technologies (Bangalore, India). Oasis MCX 1 cc (30 mg) extraction cartridges were obtained from Waters (Bangalore, India). CH_2_Cl_2_, EtOAc, MeOH, and other sample preparation reagents were purchased either from Sigma Aldrich, Fisher scientific or Sisco Research Laboratories (Mumbai, India).

### Urine sample collection

Single-void urine samples used in this study were collected as part of an ongoing research that aims to characterize tobacco exposures and cancer risk in SLT users in Mumbai, India. Non-users of any tobacco products were recruited among staff workers at the ACTREC. Users of SLT were recruited among healthy individuals (friends and relatives) who accompanied patients visiting Head and Neck Cancer Clinic at Tata Memorial Hospital in Mumbai. For analyses reported here, we selected urine samples from those SLT users who reported regular use of any SLT product for more than 5 years, were current users (within the last 24 h), reported not smoking a combustible product such as cigarettes or bidis, and reported being in generally good health. Urine was collected by a standard “clean-catch” technique into 30-mL collection cups, placed in a portable cooler with ice-packs until same-day delivery to the laboratory at ACTREC, and stored in the laboratory at − 20 °C until analyses.

Subject recruitment and sample collection were approved by the Institutional Ethics Committee of ACTREC, Tata Memorial Centre (study number 900095). The study was carried out in accordance with the Declaration of Helsinki and International Conference on Harmonization-Good Clinical Practice guidelines. All participants were adults over 18 years of age, and provided informed consent.

### Sample preparation

Sample preparation procedure included 4 key steps: (1) treatment with *β*-glucuronidase to cleave free NNAL, NNN, and cotinine from their glucuronides; (2) supported liquid extraction on Chem Elut cartridges; (3) solid-phase extraction by mixed phase cation exchange on Oasis MCX cartridges; and (4) solid-phase extraction by normal-phase Bond Elut Silica cartridges. Steps (1)–(3) were performed essentially as previously described, with minor modifications^21–23,25^. Briefly, 2 mL of urine sample (thawed at room temperature) was mixed with 1.5 mL 30 mM KH_2_PO_4_ buffer (pH 7), a mixture of isotope-labeled internal standards was added (100 pg of ^13^C_6_-NNN, 200 pg of ^13^C_6_-NNAL and 200 ng of [D_3_]cotinine per sample), and samples were treated with 15,000 units *β*-glucuronidase overnight at 37 °C. Next day, samples were purified on Chem Elut cartridges, with the eluants (2 × 8 mL of CH_2_Cl_2_) being collected into tubes containing 20 µL of ascorbic acid (1.2 µmol/µL) to prevent artefactual formation of NNN^22,23,30^. The eluants were dried (SpeedVac vacuum concentrator, Thermo Scientific) reconstituted in 500 µL water, and acidified with 50 µL of 1 N HCl. Ascorbic acid was added again, and samples were loaded on 30 mg Oasis MCX SPE cartridges and purified as described^21,25^. Additional detail can be found in Supplemental Figure S1. In step (4), the normal-phase extraction procedure that is used in sample purification for NNN analysis^23,25^ was modified so that NNAL and cotinine can be eluted together with NNN. Samples dried after the MCX purification were re-dissolved in 500 µL of CH_2_Cl_2_, loaded on 100 mg Bond Elut cartridges pre-equilibrated with 1 mL CH_2_Cl_2_, and the cartridges were then washed with 1 mL CH_2_Cl_2_ and 1 mL CH_2_Cl_2_:EtOAc (50:50) sequentially, with the eluants going to waste. The analytes were then eluted with 3 mL EtOAc:MeOH (90:10). The collected eluants were dried and transferred to glass LC microinsert vials with two 100-µL volumes of ACN. The transferred samples were dried and stored at − 20 °C until analysis. At the time of analysis samples were re-dissolved 25 µL deionized H_2_O and 10 µL were injected into the LC–MS/MS system.

### Biomarker analysis by LC–MS/MS

LC–MS/MS analysis was performed on a Shimadzu Nexera X2 ultra performance liquid chromatography system (Japan) coupled with an AB Sciex QTRAP-4500 (USA) system comprising of Turbo V™ source with ESI probe. Chromatographic separation was achieved on a Zorbax SB C18, 1.8 µ, 3.0 × 150 mm (Agilent Technologies) column eluted in gradient mode with a flow rate of 0.4 mL/min. Column temperature was maintained at 40 °C. 15 mM ammonium acetate (A) and 100% MeOH (B) were used as mobile phase for elution. Gradient was maintained at 5% B for initial three minutes, which was gradually increased to 70% B from 3 to 10 min. Composition of B was then decreased to 40% from 10 to 12 min and maintained at 40% for next three minutes. Column was re-equilibrated by returning to 5% B for 5 min before next run.

MS/MS detection was conducted by electrospray ionization in the positive ion mode using multiple reaction monitoring (MRM). Ion source parameters were optimized and applied as follows: mass spectrometer ion spray voltage of 5500 V; source temperature 500 °C; nebulizer gas and heater gas were set at 50 psi and curtain gas at 40 psi. Collision energy of 32 V was applied for cotinine analysis and 14.5 V for NNAL and NNN. Resolution of the quadrupoles (Q1 and Q3) was set at unit mass. MRM transitions monitored for quantitative analysis are listed in Table 3. To overcome the wide differences in typical urinary concentration ranges between cotinine and the other two biomarkers, we monitored ^13^C cotinine, which naturally occurs in urine, as previously described^21^.

**Table 3:** MRM transitions of NNAL, NNN and cotinine and their corresponding isotope-labeled internal standards.

Analyte	MRM Transition	
	Precursor ion (m/z)	Product ion (m/z)
NNAL	210.1	180.1
^13^C_6_-NNAL	216.1	186.1
NNN	178.1	148.1
^13^C_6_-NNN	184.1	154.1
^13^C-Cotinine	178.1	98.1
^13^C-[D_3_]Cotinine	181.1	101.1

### Method characterization

Accuracy of the assay was determined by adding different amounts of NNAL, NNN, and cotinine to pooled urine from several non-tobacco users and performing the assay as described above. The levels of added biomarkers were in the range and at ratios typically reported for tobacco users:^21,22,24^ NNAL was added at 0.1, 0.5, 2.4, 4.8 and 23.9 pmol/mL urine, NNN was added at 0.06, 0.3, 1.4, 2.8 and 5.6 pmol/mL urine, and cotinine at 0.6, 5.6, 28.2, 56.5 and 84.7 nmol/mL urine. At each level, five replicates were analyzed and precision was assessed as % CV. Intra-day and inter-day variation were determined by analyzing positive control urine samples (pooled smokers’ urine with no biomarkers added to it) on two separate days. Calculations of the S/N ratios for the determination of LOQ and in the analyses of urine samples from SLT users were performed in analyst software version 1.7.1.

### Analyses of urine samples from SLT users

Urine samples from SLT users were analyzed by following the purification and LC–MS/MS analysis procedures described above. Quantitation of biomarkers was based on the analyte-to-internal standard ratio, using calibration curves constructed with variable levels of each biomarker (in the range of values reported in Table 1) and constant levels of the corresponding internal standards: 10 pg/µL for ^13^C_6_-NNAL and ^13^C_6_-NNN and 10 ng/µL for [D_3_]cotinine. Because of the complexity and inter-individual variation of urine matrix, S/N ratios were monitored to ensure that the values are above 5 (the LOQ parameter) for the reported values.

## Supplementary Information

Supplementary Information.
