# Supplementary material for: Simultaneous analysis of urinary total 4-(methylnitrosamino)-1-(3-pyridyl)-1-butanol, N′-nitrosonornicotine, and cotinine by liquid chromatography-tandem mass-spectrometry
Source: Sci Rep. 2021 Oct 8;11:20007. doi: 10.1038/s41598-021-99259-z (PMC8501032; doi:10.1038/s41598-021-99259-z)

**Simultaneous Analysis of Urinary Total 4-(Methylnitrosamino)-1-(3-pyridyl)-1-butanol, *N*′-Nitrosonornicotine, and Cotinine by Liquid Chromatography-Tandem Mass-Spectrometry**

Sampada S. Nikam, Murari Gurjar, Hitesh Singhavi, Anand Patil, Arjun Singh, Peter Villalta, Pankaj Chaturvedi, Samir S. Khariwala, Vikram Gota, Irina Stepanov

**Supplementary Materials**

**Table S1**: Accuracy and precision of the assay at added levels of NNAL, NNN and Cotinine in pooled non-tobacco users’ urine

| **Biomarker** | **Added conc.** | **Measured conc.** | **Accuracy (%)** | **Precision (%)** |
| --- | --- | --- | --- | --- |
| NNAL(pmol/mL) | 0.12 | 0.13 | 107.5 | 4.2 |
|  | 0.48 | 0.49 | 102.5 | 7.2 |
|  | 2.39 | 2.73 | 114.1 | 1.2 |
|  | 4.79 | 5.50 | 115.0 | 1.3 |
|  | 23.9 | 27.6 | 115.3 | 0.5 |
| NNN (pmol/mL) | 0.06 | 0.05 | 96.4 | 13.8 |
|  | 0.28 | 0.24 | 85.1 | 3.6 |
|  | 1.41 | 1.27 | 90.0 | 3.1 |
|  | 2.83 | 2.48 | 87.8 | 1.5 |
|  | 5.65 | 4.91 | 86.8 | 2.4 |
| Cotinine (nmol/mL) | 0.57 | 0.64 | 113.3 | 2.6 |
|  | 5.65 | 6.18 | 109.4 | 0.8 |
|  | 28.3 | 29.7 | 105.3 | 1.9 |
|  | 56.5 | 59.1 | 104.7 | 1.8 |
|  | 84.8 | 87.7 | 103.5 | 2.3 |

**Figure S1:** Flow chart for sample preparation protocol for simultaneous analysis of urinary NNAL, NNN and cotinine


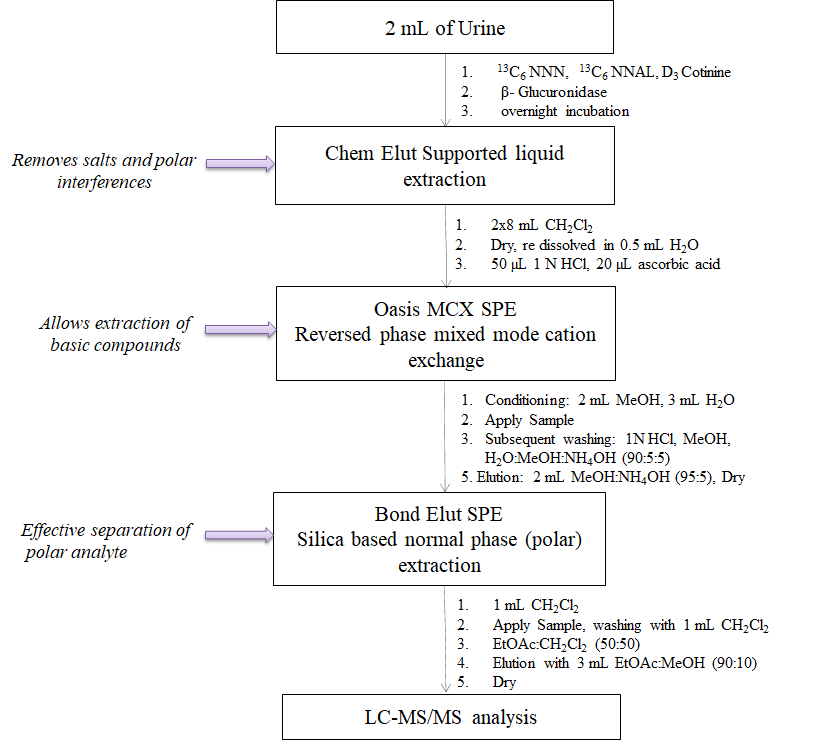

Supplement: Supplementary file 1 — Supplementary Information. [file 41598_2021_99259_MOESM1_ESM.docx]
